# Social responsibility and subjective well-being of volunteers for COVID-19: The mediating role of job involvement

**DOI:** 10.3389/fpsyg.2022.985728

**Published:** 2022-10-26

**Authors:** Chao Wu, Sizhe Cheng, Yinjuan Zhang, Jiaran Yan, Chunyan He, Zhen Sa, Jing Wu, Yawei Lin, Chunni Heng, Xiangni Su, Hongjuan Lang

**Affiliations:** ^1^School of Nursing, Air Force Medical University, Xi’an, Shaanxi, China; ^2^Department of Military Medical Psychology, Air Force Medical University, Xi’an, Shaanxi, China; ^3^Medical Department, 69245 Troop of the Chinese People’s Liberation Army, Xinjiang, China; ^4^Tangdu Hospital, The Second Affiliated Hospital of Air Force Military Medical University, Xi’an, Shaanxi, China

**Keywords:** volunteers for COVID-19, social responsibility, subjective well-being, job involvement, structural equation model

## Abstract

**Aim:**

Our study aimed to investigate the effect of social responsibility on the subjective well-being of volunteers for COVID-19 and to examine the mediating role of job involvement in this relationship.

**Background:**

Nowadays, more and more people join volunteer service activities. As we all know, volunteer work contributes to society without any return. Volunteers often have a strong sense of social responsibility and reap subjective well-being in their dedication. Although research shows that social responsibility will drive them to participate in volunteer work actively, it is less clear whether job involvement will impact their subjective well-being.

**Methods:**

The data were collected in the precaution zone in Shanghai, China, from April to May 2022. A sample of 302 volunteers for COVID-19 completed the social responsibility scale, subjective well-being scale and job involvement scale in the form of an electronic questionnaire on their mobile phones. A structural equation model was adopted to verify the research hypotheses.

**Results:**

Social responsibility was significantly and positively related to volunteers’ subjective well-being and job involvement (*p* < 0.05). Job involvement fully mediates the relationship between volunteers’ social responsibility and subjective well-being.

**Conclusion:**

Social responsibility is critical to predicting volunteers’ subjective well-being. Job involvement plays an intervening mechanism in explaining how social responsibility promotes volunteers’ subjective well-being.

## Introduction

At present, COVID-19 is still spreading all over the world, seriously hindering social development and changing people’s daily life (de Figueiredo et al., 2021; Thomson et al., 2022). The new coronavirus continues to be rampant in some areas of China, and the Chinese government has taken static management over these areas to manage the epidemic (Liu et al., 2021; Zheng et al., 2022). In the static control area in China, residents responded to the call of self-conscious isolation and stayed at home (Xu et al., 2021; Hong et al., 2022).

Medical workers need to enter the control area for nucleic acid testing and patient treatment, and the volunteer team spontaneously established by residents to cooperate with medical workers to maintain order and distribute daily supplies to residents (Chung et al., 2021; Karki et al., 2021). Although volunteer work contributes to society without any return, many people are still willing to participate in this trend (Micheel, 2017; Willems et al., 2021). In recent years, volunteerism has been widely spread among Chinese people, especially young people (Zhang et al., 2021; Yang et al., 2022). Voluntary service greatly benefits people in need and society (Liu et al., 2020; Bailey and Kaplan, 2022). Taking the prevention and control of the outbreak of pneumonia as an example, hundreds of thousands of volunteers have played an active role in the COVID-19-stricken areas. They have contributed daily necessities and provided psychological help for resident relief.

Subjective well-being refers to the individual’s cognition and evaluation of self-well-being and quality of life (Anglim et al., 2020). When volunteers do volunteer work, they will be exposed to a new environment where they can learn how to work in a team and improve their interpersonal and organizational skills. They realize their value in helping others (Serrat-Graboleda et al., 2021). Volunteer service can enhance individual happiness and subjective well-being (Wang et al., 2022). Volunteer service can significantly improve the individual’s physical and mental health, coping style and life satisfaction guided by “altruism” (Powell et al., 2021). In voluntary service, their values will be realized, enhancing their satisfaction with life and life, thus reducing their attention to negative aspects (Tse, 2020).

Social responsibility refers to the individual’s ethical care and obligation to others and society (Ahola-Launonen, 2016; Gay, 2021). Volunteers participate in volunteer activities because of their sense of social responsibility, which drives them to do what is beneficial to society (Brusa and Barilan, 2021). They expressed their duties and obligations to the community with actions (Wray-Lake et al., 2016). Bielefeldt and Canney (2016) found that social responsibility was closely related to voluntary activities, and the decline in individuals’ sense of social responsibility will make them more likely to have decreased the frequency that they participate in volunteer activities.

In addition, we attempted to explore the mechanisms through which social responsibility enhances volunteers’ subjective well-being. Previous studies have shown that social responsibility is closely related to job involvement (Glavas, 2016). It is because, with a sense of responsibility to society, individuals will actively participate in their work and have higher work enthusiasm, rather than neglect their duties. Job involvement is also positively correlated with subjective well-being (Hafner et al., 2020; Zalewska, 2020). Research showed that there was a significant relationship between employees’ subjective well-being, social support, supervisor support and job involvement. Job involvement will impact employees, which cannot only make them feel satisfied in the workplace but also generate happiness outside of work (Shiri et al., 2020). Volunteers for COVID-19 are non-professionals fighting at the epidemic’s front line, and their volunteer activities are dangerous. This group should also receive positive attention. We believe that subjective well-being, job involvement and social responsibility are related, and job involvement may serve as an intervening mechanism between volunteers’ social responsibility and subjective well-being. Hence, our study aims to investigate the effect of social responsibility on volunteers’ subjective well-being and explore the mediating role of job involvement among volunteers for COVID-19. As such, we attempted to shed light on the antecedents of volunteers’ subjective well-being from the perspective of volunteers’ social responsibility and explore under what mechanisms could this influence process happened. Therefore, carrying out social responsibility-related research on volunteers for COVID-19 is of great significance for stabilizing and expanding the volunteer team to help them get a better sense of social experience.

## Literature review and hypotheses

### Social responsibility and subjective well-being

A person with a sense of social responsibility should have three main qualities: Firstly, adhere to morally correct ideas; Secondly, comply with the principle of practical justice; At the same time, willing to make dedication and sacrifice for others (Hawkes et al., 2020; Janeway et al., 2022). Studies investigating the relationship between social responsibility and well-being revealed that good social responsibility was closely related to well-being (Macassa et al., 2021). Subjective well-being is a kind of well-being that is the degree of individual self-perception of well-being (Toh et al., 2020). When an individual has a good sense of social responsibility, his perception and view of society are more positive. At the same time, he is more willing to undertake social responsibility that does not ask for a return, so it is easier for him to be satisfied emotionally (Anderson et al., 2021). Therefore, based on these arguments, our first hypothesis is:

*H1*: Social responsibility is positively and directly related to volunteers’ subjective well-being.

### Job involvement as a mediator

Job involvement refers to the psychological recognition of work and regards work performance as the reflection of a person’s values (Calvo et al., 2021). Volunteers are spontaneous and active in voluntary activities, so they have high work enthusiasm and engagement in their work (Mullan et al., 2021). Research shows that intention and habit strength are significant predictors of volunteering engagement among volunteers (Mullan et al., 2021), and this intention mainly comes from volunteers’ sense of social responsibility. The work of volunteers reflects the social responsibility of being willing to serve others and the public. Currently, social responsibility and job involvement research is mostly focused on enterprises and employees (Ahmad et al., 2021). Seivwright and Unsworth (2016) found that corporate social responsibility can be a driving force behind employees’ job involvement. Social responsibility and job involvement are closely related (Farid et al., 2019).

Volunteers engage in voluntary activities out of their sense of responsibility to society (Klug et al., 2018). Volunteer service can significantly improve volunteers’ expression, planning, communication, action, team spirit and empathy (Wu et al., 2019). The improvement of individual ability can help to enhance subjective well-being. At the same time, a higher social evaluation of volunteers and social responsibility can bring them positive cognitive styles and emotions and help improve volunteers’ sense of self-efficacy (Dionigi et al., 2020). Self-efficacy is one of the important factors that affect subjective well-being (Chudzicka-Czupała and Zalewska-Łunkiewicz, 2020; Bhattarai et al., 2021). Research shows that people with a good sense of social responsibility often have a good sense of self-efficacy (Wang et al., 2021), and the improvement of self-efficacy can help individuals gain more confidence and face difficulties and problems with a positive attitude (Gallagher et al., 2020).

Job involvement reflects a working state, while subjective well-being demonstrates an individual’s evaluation of all aspects of life. Research shows that job involvement can positively predict subjective well-being (Engelbrecht et al., 2020). For example, Hakanen and Schaufeli (2012) used structural equation modeling to investigate the relationship between job involvement and subjective well-being. The results show that job involvement is manifested in employees’ happy emotions and satisfied and highly activated states. These positive work experiences help to improve employees’ subjective well-being (Hakanen and Schaufeli, 2012).

We thus expect that job involvement would mediate the association between social responsibility and volunteers’ subjective well-being. A good sense of social responsibility can make volunteers more actively participate in volunteer activities. At the same time, job involvement can help them gain many benefits in volunteer work to improve their subjective well-being. Taken together, we propose that:

*H2*: Job involvement mediates the relationship between social responsibility and volunteers’ subjective well-being.

## Materials and methods

### Participants and data collection

Participants were volunteers from some enclosed communities in Shanghai from April to May 2022. They were residents of the community, some were students, some were workers, some were retirees, and people from all walks of life. They spontaneously formed a volunteer team to assist the management of the lockdown zone managers. With the community’s help, the questionnaires were distributed electronically to these volunteers through mobile phones. The inclusion criteria were volunteers who assisted in community management in the lockdown zones and voluntarily participated in the survey. The questionnaire was completed under the guidance of unified guidelines and we obtained their informed consent before starting the formal investigation. They were informed that they could withdraw from the study at any time for any reason. The questionnaire filling time shall not be less than 10 min in the electronic system to ensure the accuracy of data collection. In addition, the questionnaires were anonymous, and the research group would protect the data to ensure that it would not be disclosed or used for other purposes. The calculation of the sample size is 5–10 times of the number of items in the questionnaire (Vanbutsele et al., 2018). There were 59 items in this questionnaire. Therefore, the calculation formula of sample size was *N* = (32 + 9 + 18) * 5 = 295, which meant that at least 295 subjects were required for this study. Finally, we surveyed a total of 313 volunteers in 11 communities. Among them, six quit the study in the middle of the survey, and five filled in the questionnaire with too high homogeneity. Therefore, we finally recovered 302 valid questionnaires, with an effective recovery rate of 96.49%.

### Measures

#### Social responsibility

Social responsibility was measured using the social responsibility scale (Olmsted and Monachesi, 1956). The scale contains 32 items and is scored based on yes or no. An example item is “I often feel remorse for my temper tantrums and complaints.” The score of the scale ranges from 0 to 32, and the higher the score of the scale, the stronger the individual’s social responsibility. The lower the score, the worse the individual’s social responsibility. The scale has good reliability and validity, and the Cronbach’s alpha coefficient of the scale was 0.913.

#### Job involvement

Job involvement was measured using the job involvement scale (Kaplan et al., 1991). The scale contains 9 items from 3 dimensions, including vitality, concentration and dedication. An example item is “I like the sense of achievement that work brings.” A 7-point Likert-type scale was adopted, with a full score of 63. Higher scores indicated higher job involvement. The scale is widely used in China and has good reliability and validity (Huang et al., 2019). The Cronbach’s α was 0.936 and ranged between 0.803 and 0.929 for the three dimensions.

#### Subjective well-being

Volunteers’ subjective well-being was measured using the subjective well-being scale (Clark et al., 2019). The scale contains 18 items from 6 dimensions, including vigor, relaxation or tension, pleasure or anxiety, self-controlled, health concerns and satisfaction. An example item is “Are you happy, content or relaxed in your life?” The scale was scored by Likert’s 6-level (item 1-item 14) and 10-level (item 15-item 18) mixed scoring method. The higher the score, the stronger the subjective well-being of the respondents. The scale widely applies to the Chinese population (Shui et al., 2020; Huang and Fang, 2021), and the Cronbach’s α was 0.921 and ranged between 0.832 and 0.940 for the six dimensions.

### Statistical analysis

The exploratory factor analysis was used to test the possible common method bias (Wingate et al., 2018). The results showed that the first common factor interpretation rate was less than the critical standard of 40%, and there was no common method bias in the data of our study. We used SPSS 26.0 statistical software and Mplus 8.3 for statistical analysis. The measurement data was expressed in the form of mean ± standard deviation. The Pearson correlation coefficient was used to analyze the correlations between social responsibility, job involvement and subjective well-being. Finally, we adopted a two-step structural equation modeling procedure to analyze the mediating effects of job involvement between volunteers’ social responsibility and subjective well-being. Then, we ran 2,000 bootstrapping resamples to examine the mediator effect of job involvement. The confidence interval is 95% and does not contain 0, which signifies statistical significance (Steinwender et al., 2020).

### Ethical approval

Our study was conducted under ethical guidelines described in the Helsinki Declaration (Sanchini et al., 2020). Since there was no unethical behavior in the study and our study did not involve human clinical trials or animal experiments, our research required no ethical approval. Before the investigation, we explained the purpose to the participants, asked them for oral consent, and signed the electronic informed consent form. During the survey, participants could terminate and withdraw from the study at any time, and the questionnaire was filled in anonymously.

## Results

### Descriptive statistics of the measurement scales

All participants were volunteers from the control area of Shanghai during COVID-19. The data were firstly treated with a normal distribution test, and correspond to normal distribution. A total of 302 valid questionnaires were collected. Respondents had an average age of 34.64 (SD = 9.80, ranging from 15 to 66). Table 1 shows the descriptive statistics of the measurement scales. The score of volunteers’ social responsibility was (23.51 ± 3.24), job involvement score was (55.93 ± 8.30), and subjective well-being score was (79.45 ± 6.95) which indicates that volunteers for COVID-19 have a high level of job involvement and a medium level of social responsibility and subjective well-being.

**Table 1:** Social responsibility, job involvement and subjective well-being of volunteers.

	Min.	Max.	Mean	SD
Social responsibility	13	31	23.51	3.24
Job involvement	9	63	55.93	8.30
Vitality	3	21	18.73	2.91
Concentration	3	21	18.90	2.82
Dedication	3	21	18.30	3.12
Subjective well-being	60	102	79.45	6.95
Vigor	10	24	17.55	2.13
Relaxation or tension	12	26	18.46	3.06
Pleasure or anxiety	8	22	18.19	3.12
Self-controlled	3	12	6.22	2.11
Health concerns	6	16	13.71	2.10
Satisfaction	2	10	5.32	1.93

### Correlational analysis of social responsibility, job involvement and subjective well-being

Table 2 shows the Pearson correlation coefficients among social responsibility, job involvement and subjective well-being of volunteers for COVID-19. The results showed that social responsibility was positively correlated with job involvement (*r* = 0.263, *p* < 0.01) and its three dimensions (*r* = 0.254, *p* < 0.01; *r* = 0.248, *p* < 0.01; *r* = 0.258, *p* < 0.01), and social responsibility was positively correlated with subjective well-being (*r* = 0.201, *p* < 0.01) and its six dimensions (*r* = 0.214, *p* < 0.01; *r* = 0.282, *p* < 0.01; *r* = 0.202, *p* < 0.01; *r* = 0.322, *p* < 0.01; *r* = 0.260, *p* < 0.01; *r* = 0.201, *p* < 0.01). In addition, job involvement was positively correlated with subjective well-being (*r* = 0.296, *p* < 0.01) and its six dimensions (*r* = 0.255, *p* < 0.01; *r* = 0.220, *p* < 0.01; *r* = 0.379, *p* < 0.01; *r* = 0.175, *p* < 0.01; *r* = 0.319, *p* < 0.01; *r* = 0.222, *p* < 0.01).

**Table 2:** Correlations among study variables.

		1	2	3	4	5	6	7	8	9	10	11
1	Social responsibility	1										
2	Job involvement	0.263^**^	1									
3	Vitality	0.254^**^	0.931^**^	1								
4	Concentration	0.248^**^	0.958^**^	0.867^**^	1							
5	Dedication	0.258^**^	0.926^**^	0.761^**^	0.835^**^	1						
6	Subjective well-being	0.201^**^	0.296^**^	0.284^**^	0.309^**^	0.244^**^	1					
7	Vigour	0.214^*^	0.255^**^	0.186^**^	0.163^**^	0.092^**^	0.681^**^	1				
8	Relaxation or tension	0.282^**^	0.220^**^	0.192^**^	0.218^**^	0.207^**^	0.725^**^	0.302^**^	1			
9	Pleasure or anxiety	0.202^**^	0.379^**^	0.413^**^	0.369^**^	0.289^**^	0.620^**^	0.379^**^	0.458^**^	1		
10	Self-controlled	0.322^**^	0.175^**^	0.211^**^	0.144^*^	0.139^*^	0.124^*^	0.150^*^	0.204^**^	0.512^**^	1	
11	Health concerns	0.260^**^	0.319^**^	0.313^**^	0.312^**^	0.274^**^	0.594^**^	0.317^**^	0.347^**^	0.460^**^	0.269^**^	1
12	Satisfaction	0.201^**^	0.222^* *^	0.266^**^	0.193^**^	0.168^**^	0.127^*^	0.130^*^	0.204^**^	0.469^**^	0.493^**^	0.299^**^

** *p* < 0.01;

* *p* < 0.05.

### Verification of the intermediary role of job involvement

Firstly, we assessed the measurement model, including three latent constructs (social responsibility, job involvement and subjective well-being). After modification, confirmatory factor analysis revealed that the three-factor model fit the data well: *χ*^2^ = 156.292, df = 46, *χ*^2^/df *=* 3.398, Comparative Fit Index (CFI) = 0.916, Tucker-Lewis Index (TLI) = 0.933, Root-Mean-Square Error of Approximation (RMSEA) = 0.040, 90% CI: 0.032–0.067, Standardized Root Mean Square Residual (SRMR) = 0.080 (*p* < 0.01), and all indicators were significantly loaded on the corresponding constructs.

**Figure 1:**
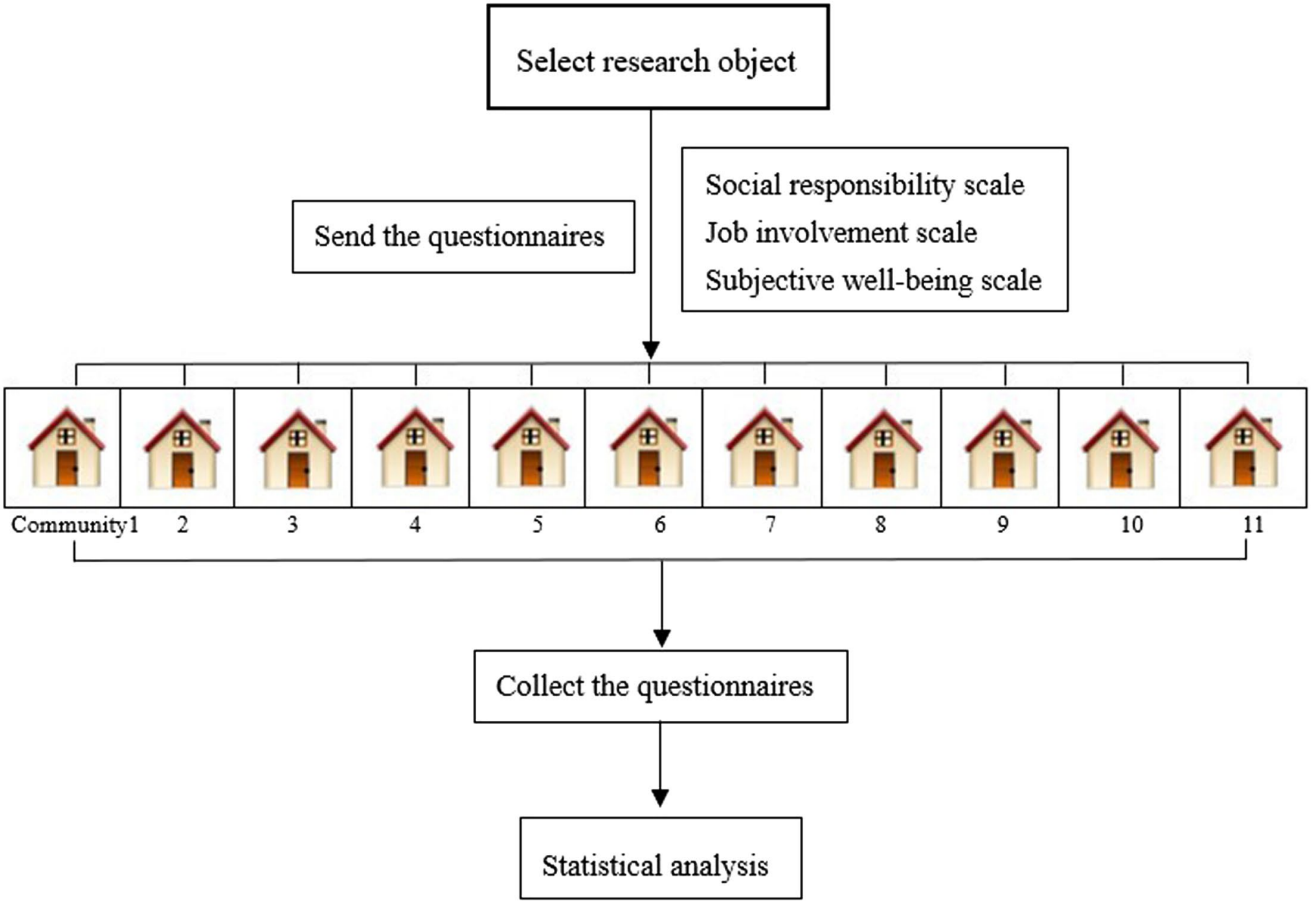
The experimental process.

Next, we tested a direct effect model to verify whether social responsibility positively affects volunteers’ subjective well-being (H1). After modification, the results showed that the direct effect model exhibited a good fit to the data: *χ*^2^ = 119.845, df = 26, *χ*^2^/df *=* 4.609, Comparative Fit Index (CFI) = 0.928, Tucker-Lewis Index (TLI) = 0.942, Root-Mean-Square Error of Approximation (RMSEA) = 0.047, 90% CI: 0.041–0.062, Standardized Root Mean Square Residual (SRMR) = 0.017 (*p* < 0.01). Figure 1 depicts the direct model. Social responsibility was positively associated with subjective well-being (*β* = 0.294, *p* < 0.01). Then we performed 2,000 bootstrapping resamples to justify the 95% CI of the total direct effect of social responsibility on the subjective well-being of volunteers for COVID-19. The bootstrapping results showed that the 95% CI for the total direct effect was (0.063, 0.525).

**Figure 2:**
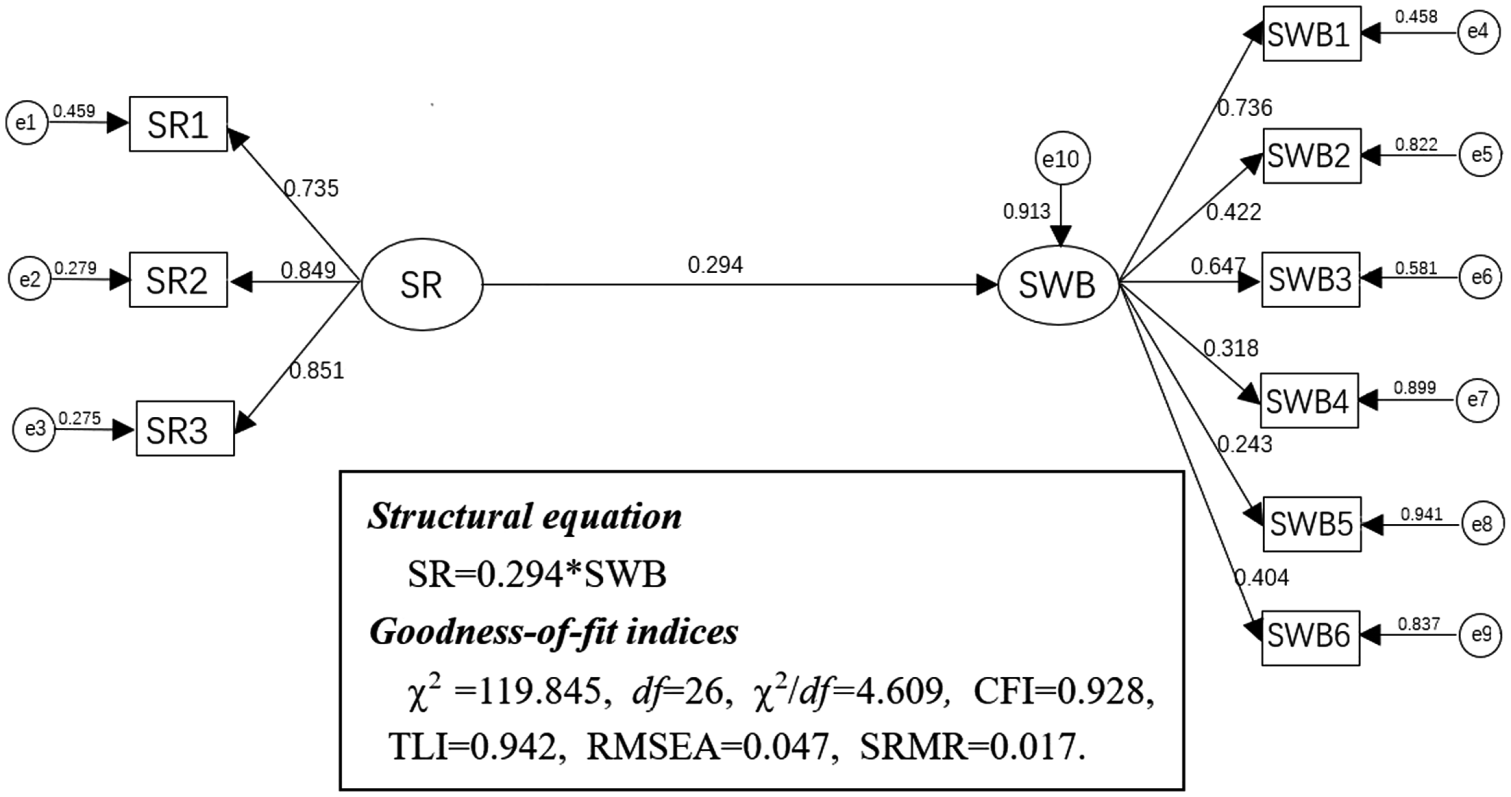
Direct effect model. SR, Social responsibility; e1–e3, manifest variables of the package of social responsibility; SWB, Subjective well-being; e4–e9, manifest variables of the six dimensions of subjective well-being.

**Figure 3:**
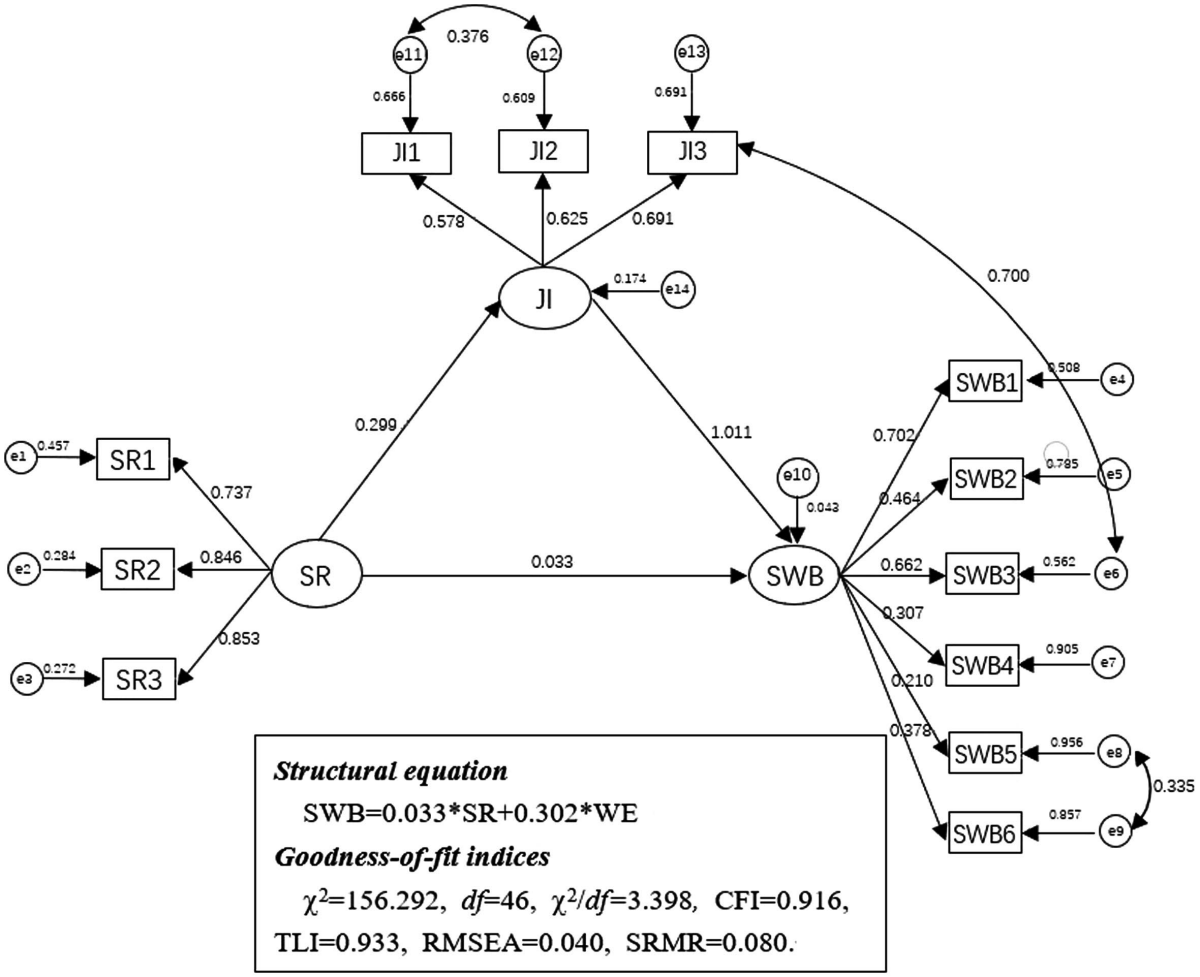
Mediation model. SR, Social responsibility; JI, Job involvement; SWB, Subjective well-being.

Finally, we tested a mediating effect model to verify whether job involvement mediates the relationship between social responsibility and subjective well-being (H2). We repeated this process 2000 times to arrive at an empirical approximation of the sampling distribution and obtained the estimate and confidence interval for this indirect effect. After model modification, according to the confirmatory factor analysis, the three-factor model had an adequate fit to the data with *χ*^2^ = 156.292, df = 46, *χ*^2^/df *=* 3.398, Comparative Fit Index (CFI) = 0.916, Tucker-Lewis Index (TLI) = 0.933, Root-Mean-Square Error of Approximation (RMSEA) = 0.040, 90% CI: 0.032–0.067, Standardized Root Mean Square Residual (SRMR) = 0.080 (*p* < 0.01), and all indicators were significantly loaded on the corresponding constructs. Figure 2 depicts the mediating effect model. Social responsibility was positively related to job involvement (*β* = 0.299, *p* < 0.01), which in turn had a positive effect on subjective well-being (*β* = 1.011, *p* < 0.01). We performed 2000 bootstrapping resamples to justify the 95% CI of the indirect effect of social responsibility on subjective well-being *via* job involvement. Table 3 showed that the results of bootstrapping showed that the 95% CI of the mediating effect was (0.099, 0.506). The total effect was 0.335, and the indirect effect was 0.302. The indirect effect accounted for 90.15% of the total effect of social responsibility on the subjective well-being of volunteers for COVID-19 (Figure 3).

**Table 3:** Confidence interval of mediating effect value in chain mediated model (2,000 bootstrap samples).

Model path	Estimate	95%CI	
		LLCI	ULCI
SR → SWB	0.294	0.063	0.525
SR → WE→SWB	0.302	0.099	0.506

LLCI, Lower Limit Confidence Interval; ULCI, Upper Limit Confidence Interval.

## Discussion

Shanghai had been closed down to control the epidemic, and the medium and high-risk areas had entered a “closed” state (Zhang et al., 2022). People responded to the call, lived in isolation, and stayed indoors (Király et al., 2020). Who killed the virus for the streets and communities? Who helped the unknown residents to buy daily necessities? It is them, volunteers. COVID-19 is highly infectious, so it is dangerous to engage in volunteer service under this background. By investigating the subjective well-being of volunteers in COVID-19, we can better understand their psychological and work status and then better carry out community management under the background of the epidemic.

To a large extent, volunteers’ participation in voluntary activities stems from their strong sense of social responsibility (Omoto and Snyder, 1993), and there is no doubt that volunteering contributes to our society and people in need. The volunteers provide their assistance without requiring any financial rewards. Their contributions have a great impact on a harmonious society contribution. Volunteering has been shown to benefit individuals directly, such as reducing depression and improving life satisfaction and well-being (Okun et al., 2016). Individuals who participate in voluntary service can get a lot of feedback, such as acquiring a certain skill, expanding interpersonal relationships, improving social participation, increasing positive self-awareness, and thus improving self-worth (Kim et al., 2020; Mo et al., 2022). However, limited studies have examined whether and how social responsibility might impact volunteers’ subjective well-being, especially in the background of COVID-19. Therefore, the objective of our study was to explore the effect of social responsibility on volunteers’ subjective well-being using the mediating mechanism of job involvement. To our knowledge, this is the first research to examine this proposition empirically.

First, our results evidence that social responsibility has a direct and positive effect on volunteers’ subjective well-being, supporting H1. Social responsibility and individual well-being are closely related (Macassa et al., 2021). Volunteers engage in voluntary service out of their sense of social responsibility. The recognition of the public in the process of voluntary service will make them emotionally happy and proud and produce a sense of satisfaction. It is an important form of subjective well-being (Robak et al., 2007; Reeskens and Wright, 2011).

Moreover, our results suggest that job involvement is an important intervening mechanism between social responsibility and subjective well-being. Our results show that job involvement partially mediates the link between social responsibility and subjective well-being. Many studies have shown that individuals’ high level of work input and job involvement, especially their dedication to work, will help them gain greater job rewards and organizational identity (Chen and Chiu, 2009; Katrinli et al., 2009). It can be in the form of material or spiritual rewards, which is an important aspect of the source of subjective well-being (Ren et al., 2021). The high work input of volunteers comes from their sense of responsibility to society. They feel that it is their duty to serve the community. Volunteers combine serving others, serving society and realizing personal values, and explaining the spirit of volunteerism with practical actions. Through voluntary service, they have recognized their self-worth in their work, that is, their dedication to society, and at the same time, they have been praised and recognized by the community, which is conducive to improving their subjective well-being (Gentry et al., 2018; Cramer et al., 2020).

Improving volunteers’ subjective well-being is conducive to stimulating their work enthusiasm and job involvement and better playing their role in the service, which is the significance of our study. Therefore, government departments and community managers should strengthen the guarantee of volunteer service and pay attention to the supervision and encouragement of volunteers. We can cultivate and reinforce volunteers’ sense of social responsibility, stimulate their work enthusiasm, and provide knowledge support, emotional support, and administrative support by organizing regular training and supervision activities to enhance volunteers’ sense of value and belonging and maintain a long-term and stable volunteer service relationship. It is necessary to establish and improve the volunteer incentive and recognition mechanism. We can standardize the assessment and incentive methods, record the service time, evaluate the service quality through observation or feedback from service objects and institutions, commend outstanding volunteers, and stimulate the enthusiasm for volunteer service and the level of work input to improve their sense of self-worth and self-efficacy and enhance subjective well-being.

## Limitations

There are some limitations to our study. First of all, due to the impact of the epidemic situation and the busy tasks of volunteers, our study did not adopt a stratified random design and only used a convenient sampling method. Although the data volume met the requirements of the minimum sample size, it was too small. Secondly, our study was conducted as a self-report questionnaire, and the results tended to be subjective. Thirdly, we conducted a cross-sectional study, and there might be some causal relationships among social responsibility, job involvment and subjective well-being when using a longitudinal dataset. In the future study, we will conduct a longitudinal survey and conduct a causal analysis among these variables.

## Conclusion

By conducting an online questionnaire survey on 302 volunteers in the Shanghai epidemic control area, we found that volunteers for COVID-19 had a high level of job involvement and a medium level of social responsibility and subjective well-being. Social responsibility was critical to predicting volunteers’ subjective well-being. Job involvement played an intervening mechanism in explaining how social responsibility promotes volunteers’ subjective well-being. Therefore, managers can enhance their subjective well-being by strengthening their sense of social responsibility and improving their job involvement.

## Data availability statement

The original contributions presented in the study are included in the article/supplementary material, further inquiries can be directed to the corresponding authors.

## Author contributions

CW, SC, and YZ contributed to the research design and writing of the paper. JY and CyH distributed the questionnaires. ZS and JW were in charge of writing the article and verifying the English version. YL analyzed the data. CnH, XS, and HL designed the research. All authors contributed to the article and approved the submitted version.

## Funding

This work was supported by the Key Research and Development Plan of Shaanxi Province: General Projects—social development field (Grant 2020SF-280).

## Conflict of interest

The authors declare that the research was conducted in the absence of any commercial or financial relationships that could be construed as a potential conflict of interest.

## Publisher’s note

All claims expressed in this article are solely those of the authors and do not necessarily represent those of their affiliated organizations, or those of the publisher, the editors and the reviewers. Any product that may be evaluated in this article, or claim that may be made by its manufacturer, is not guaranteed or endorsed by the publisher.
